# Dissecting the interactions of SERRATE with RNA and DICER-LIKE 1 in Arabidopsis microRNA precursor processing

**DOI:** 10.1093/nar/gkt667

**Published:** 2013-08-05

**Authors:** Yuji Iwata, Masateru Takahashi, Nina V. Fedoroff, Samir M. Hamdan

**Affiliations:** ^1^Division of Biological and Environmental Sciences and Engineering, King Abdullah University of Science and Technology, Thuwal 23955-6900, Kingdom of Saudi Arabia and ^2^Center for Desert Agriculture, King Abdullah University of Science and Technology, Thuwal 23955-6900, Kingdom of Saudi Arabia

## Abstract

Efficient and precise microRNA (miRNA) biogenesis in Arabidopsis is mediated by the RNaseIII-family enzyme DICER-LIKE 1 (DCL1), double-stranded RNA-binding protein HYPONASTIC LEAVES 1 and the zinc-finger (ZnF) domain-containing protein SERRATE (SE). In the present study, we examined primary miRNA precursor (pri-miRNA) processing by highly purified recombinant DCL1 and SE proteins and found that SE is integral to pri-miRNA processing by DCL1. SE stimulates DCL1 cleavage of the pri-miRNA in an ionic strength-dependent manner. SE uses its N-terminal domain to bind to RNA and requires both N-terminal and ZnF domains to bind to DCL1. However, when DCL1 is bound to RNA, the interaction with the ZnF domain of SE becomes indispensible and stimulates the activity of DCL1 without requiring SE binding to RNA. Our results suggest that the interactions among SE, DCL1 and RNA are a potential point for regulating pri-miRNA processing.

## INTRODUCTION

microRNAs (miRNAs) are small RNAs that regulate gene expression in eukaryotic cells, playing important regulatory roles in many biological processes (1). miRNAs are transcribed by RNA polymerase II as a precursor RNA, termed the primary miRNA (pri-miRNA), which contains a hairpin structure with number of bulges and that is capped and polyadenylated at the 5′ and 3′ ends, respectively. Pri-miRNA is processed by an RNaseIII family enzyme to release the miRNA/miRNA* duplex located within the hairpin. The miRNA strand is incorporated into Argonaute protein to form the RNA-induced silencing complex that binds to target mRNAs and represses gene expression through either mRNA cleavage or inhibition of translation (1).

In plants, the RNaseIII enzyme DICER-LIKE 1 (DCL1) processes the pri-miRNA in the nucleus in two steps (1). In the first step, the pri-miRNA is cleaved to release pre-miRNA, a fold-back structure that contains the miRNA sequence at one end. In the second step, pre-miRNA is cleaved to release the miRNA/miRNA* duplex. This is in contrast to animals in which the two steps are mediated by two different RNAseIII-family enzymes, Drosha and Dicer, and take place in the nucleus and the cytoplasm, respectively (2). Several lines of evidence from *Arabidopsis thaliana* suggest that a number of proteins act together with DCL1 to ensure efficient and precise miRNA biogenesis. These include HYPONASTIC LEAVES 1 (HYL1), a double-stranded RNA-binding protein (dsRBD) containing two dsRBDs, and SERRATE (SE), a zinc-finger (ZnF) domain-containing protein. *hyl1-* and *se*-mutant plants accumulate less miRNA and more pri-miRNA than do wild-type plants (3–6). Bimolecular fluorescence complementation assays show that DCL1, HYL1 and SE co-localize in subnuclear regions termed dicing bodies or D-bodies, in which pri-miRNA processing is believed to take place (7,8). DCL1 and HYL1 co-immunoprecipitate when transiently expressed in *Nicotiana benthamiana* (9) and SE and HYL1 interact in a yeast two-hybrid system (5). Besides HYL1 and SE, there are some other proteins that have been proposed to function during miRNA biogenesis (10–17). The forkhead-associated domain-containing protein DAWDLE was found to co-immunoprecipate with DCL1 and has been proposed to facilitate DCL1 access to or recognition of pri-miRNA (11). The cap-binding protein complex (CBC) interacts with the 5′ cap structure of pri-miRNA and has been proposed to facilitate the loading of pri-miRNA processing machinery onto pri-miRNA (10,12). HUA ENHANCER 1 methylates miRNA/miRNA* duplexes, protecting them from degradation (13,15). HASTY, a plant ortholog of exportin-5, is required for miRNA to be exported from the nucleus to the cytoplasm (14).

Pri-miRNA processing is highly accurate *in vivo* in both animals and plants (18–21). The first cleavage site determines this accuracy. The second cleavage is believed to take place at a position determined by the distance between the 3′ end of pre-miRNA recognized by the Piwi/Argonaute/Zwille (PAZ) domain and the catalytic site of the RNaseIII domains. In animals, the distance from the forked-single stranded RNA (ssRNA) and the miRNA/miRNA* within the pri-miRNA-containing stem-loop structure is constant relative to the first cleavage site (11 nt), and the size of the stem loop itself is also constant (18). It is therefore easy to envision a mechanism by which Drosha, in conjunction with the dsRNA-binding protein DGCR8, recognizes a particular structure to mark the first cleavage site (18). In plants, the miRNA/miRNA* is often embedded in a longer precursor at a position 15–17 nt away from either a bulge in the duplex or the end of the duplex ssRNA (19–21), and it is believed that DCL1 uses this bulge structure to determine the first cleavage site. However, the length and the structure of the dsRNA segment between the bulge or fork and the miRNA/miRNA* varies considerably and in some plant pri-miRNAs, the first cleavage is at the end of the duplex adjacent to the terminal loop of the hairpin (22,23). The variability in the pri-miRNA structure and the differences in the orientation of the initial cleavage with respect to the terminal loop make it difficult to understand how DCL1 identifies the position of the initial cleavage with such precision *in vivo*.

DCL1 is a 214 kDa protein that consists of a DExD/H-box RNA helicase domain, a DUF283 domain, which adopts a dsRBD-like fold, a PAZ domain, which binds the 3′ end of ssRNA, two tandem RNaseIII domains and two tandem dsRBDs. The importance of the helicase and the second dsRBD has been inferred from genetic evidence that miRNA accumulation is dramatically reduced by mutation in the helicase domain or disruption in the second dsRBD in *dcl1-7* and *dcl1-9* mutants, respectively (24,25). HYL1 is a 43 kDa protein and contains two tandem dsRBDs at the N-terminus followed by the C-terminal regions consisting of six consecutive copies of a 28 amino acid repeat. It has been shown *in vitro* that HYL1 homodimerizes through its second dsRBD; this forms a non-canonical dsRBD fold that creates a dimerization interface. HYL1 binds to dsRNA via its first dsRBD (26). The C-terminal repeats of HYL1 seem to be dispensable for its function since expression of a truncated HYL1, which contains the two dsRBDs, but lacks the C-terminal repeats, fully complements the null *hyl1* mutants *in vivo* (27). SE is a ZnF domain-containing protein that is conserved in both animals and plants. Arabidopsis SE is an 81 kDa protein (720 amino acids) and contains a C_2_H_2_-type ZnF domain. The truncated SE (amino acids 194–543), termed the SE core, adopts a walking man-like topology with the ZnF domain resembling that of an isopentenyltransferase that binds to dsRNA (28).

Initial results from *in vitro* reconstituted pri-miRNA processing reactions using recombinant DCL1, HYL1 and SE proteins showed that DCL1 alone is able to cleave pri-miRNA to release the miRNA/miRNA* duplex, albeit somewhat inaccurately (29). Both HYL1 and SE were reported to stimulate cleavage of the precursor by DCL1 and to improve its accuracy (29). In the present study using highly purified proteins, we found that although DCL1 alone cleaves pri-miRNA, SE substantially enhances processing of the pri-miRNA under optimized reaction conditions. We show that SE interacts directly with DCL1 and RNA. We further analyzed binding of truncated SE variants to RNA and to DCL1. We find that SE primarily interacts with RNA through its conserved N-terminal proline/arginine-rich domain. Both the N-terminal region and the conserved ZnF domain of SE are required for its interaction with DCL1 protein. SE lacking the N-terminal sequence can bind to RNA-bound DCL1 and stimulate processing, but SE lacking its ZnF domain cannot. Thus, although the N-terminal domain of SE appears to be required for RNA binding, the ability of the N-terminally truncated SE protein to stimulate DCL1 activity suggests that it does so directly and that RNA binding is not required for this stimulation.

## MATERIALS AND METHODS

### Plasmid and bacmid preparation

We constructed plasmids to express DCL1 and SE from *A. thaliana* as an N-terminal SUMOstar-tagged form also containing both a hexahistidine tag and a Strep-tag II at the N terminus. The cDNA fragment corresponding to DCL1 and SE was amplified by PCR using primers (5′-TGCAGGAAGACAGAGGTATGGTAATGGAGGATGAGCCTAGAG-3′ and 5′-TGCAGTCTAGATCAAGAAAAAGTTTTATTTAAAAGCTCAAG-3′ for DCL1, 5′-TGCAGGGTCTCTAGGTATGGCCGATGTTAATCTTCCTCCG-3′ and 5′-TGCAGTCTAGACTACAAGCTCCTGTAATCAATAACGG-3′ for SE; restriction enzyme sites underlined). For DCL1, a silent mutation was introduced by overlapping PCR using primers (5′-GAGAAAGGTCTACTTGTTGAAGCT-3′ and 5′-AGCTTCAACAAGTAGACCTTTCTC-3′). The amplified fragments were digested by restriction enzymes (BbsI and XbaI for DCL1 and BsaI and XbaI for SE) and inserted into a BsmBI-digested pI-Strep-SUMOstar vector, which was created by inserting the Strep-tag II sequence between the hexahistidine and SUMOstar tags in the pI-SUMOstar vector (Lifesensors). For the N-terminally hexahistidine-tagged wild-type and mutant SE proteins, SE cDNA was PCR amplified using primers (5′-CACCATGGCCGATGTTAATCTTCCTCCG-3′ and 5′-TGCAGTCTAGACTACAAGCTCCTGTAATCAATAACGG-3′) and cloned into the modified pENTR-D/TOPO vector (Life Technologies). A nucleotide sequence containing myc tag and Strep tag II was inserted in the NotI site using primers (5′-GGCCGAACAAAAACTCATCTCAGAAGAGGATCTGGCGAGCGCGTGGAGCCACCCGCAGTTCGAAAAAATATCCCG-3′ and 5′-GGCCCGGGATATTTTTTCGAACTGCGGGTGGCTCCACGCGCTCGCCAGATCCTCTTCTGAGATGAGTTTTTGTTC-3′). The fragment was then inserted into the pDEST10 vector (Invtrogen). The SE deletion constructs were created by PCR amplification of the entire plasmid without the nucleotide sequences encoding the N-terminal (SE_143__–720_), C-terminal (SE_1__–701_), or ZnF domains (SE_Δ497__–547_), which were deleted using primers (5′-GACCATGGTGGAGGATATGACCGTG-3′ and 5′-ACCTCCAATCTGTTCGCGGTGAGCCTC-3′ for SE_143__–720_, 5′-TAGCTAGAGGATCCGAATTCGAGCTCC-3′ and 5′-GCTGCGCAGCCGTCTAGGATCTTGTC-3′ for SE_1__–701_, 5′-GTTGGAAATATGGTAGTGGAGCCAAGGGCAGCACGAAGCTGTTTC-3′ and 5′-GAAACAGCTTCGTGCTGCCCTTGGCTCCACTACCATATTTCCAAC-3′ for SE_Δ497__–547_) followed by self-ligation. The resulting vectors were transformed into DH10Bac (Life Technologies) to prepare bacmid DNA.

### Recombinant protein expression and purification using the baculovirus/insect cell expression system

Sf9 insect cells were cultured at 27°C in Sf-900 III SFM (Life Technologies). Bacmid DNA was transfected to Sf9 insect cells using Cellfectin II (Life Technologies) according to the manufacturer’s instructions, and the resulting supernatant was obtained as the P1 virus stock. It was then amplified to obtain a P2 virus stock, which was further amplified to obtain a P3 virus stock for large-scale expression.

For SUMOstar-DCL1, the P3 virus stock was added to 2 × 10^9^ cells and collected after 48 h. For SUMOstar-SE, the P3 virus stock was added to 5 × 10^8^ cells and collected after 24 h. After collection by centrifugation at 500 g for 10 min, the cell pellet was resuspended in 1 ml per 2 × 10^7^ cells of Buffer A [20 mM Tris–HCl (pH 7.0,) 300 mM NaCl, 20 mM imidazole, 2 mM &nbsp;β-ME] and disrupted by sonication. After removing cell debris by centrifugation, the supernatant was added to 5 ml of Ni-NTA Agarose (Qiagen) equilibrated with Buffer A and incubated for 30 min. The resin was washed five times with 40 ml of Buffer A, and the bound proteins were eluted with 20 ml of Buffer B [20 mM Tris–HCl (pH 7.0), 300 mM NaCl, 500 mM Imidazole, 2 mM β-ME]. The eluate was loaded directly onto the StrepTrap HP 1 ml (GE Healthcare), and the bound proteins were eluted with Buffer C [20 mM Tris–HCl (pH 7.0), 300 mM NaCl, 2.5 mM d-desthiobiotin, 2 mM β-ME]. The peak fractions were collected, and the SUMOstar tag was removed by SUMOstar protease (Lifesensors) digestion to generate the native DCL1 and SE. These were passed through Ni-NTA Agarose, and the untagged proteins were collected in the flow-through fractions. These were further loaded on the HiLoad Superdex 200 (GE Healthcare) and dialyzed against Buffer D [20 mM Tris–HCl (pH 7.0), 100 mM NaCl, 2 mM β-ME]. The protein concentration was determined by absorbance at 280 nm with the extinction coefficient and molecular weight calculated based on the amino acid sequence of each protein.

For hexahistidine-tagged SE wild-type and mutant proteins, P3 virus stock was added to an Sf9 cell suspension and collected after 24 h. The cell pellet was resuspended in 1 ml of Buffer E [20 mM Tris–HCl (pH 7.0), 300 mM NaCl, 5 mM imidazole, 2 mM β-ME and 20% glycerol] per 2 × 10^7^ cells and disrupted by sonication. After removal of cell debris by centrifugation, the supernatant was added to 1 ml of TALON Metal Affinity Resin (Takara) equilibrated with Buffer E and incubated for 30 min. The resin was washed three times with 10 ml of Buffer E, and the bound proteins were eluted with 2.5 ml of Buffer F [20 mM Tris–HCl (pH 7.0), 300 mM NaCl, 150 mM Imidazole, 2 mM β-ME and 20% glycerol]. The elution fractions were further loaded on the HiLoad Superdex 200 (GE Healthcare) equilibrated with Buffer F. The protein concentration was determined by the band intensity in the Coomassie blue-stained SDS–PAGE gel with a known amount of untagged SE protein resolved on the same gel.

### RNA preparation

pri-miRNA transcripts were synthesized by *in vitro* transcription by T7 RNA polymerase by using MEGAshorscript (Life Technologies) according to the manufacturer’s instructions. The DNA template was PCR amplified with primers 5′-AATTTAATACGACTCACTATAGGGATTTCTCCACTTCTTGAGCTTCC-3′ and 5′-AGTCAACTGTGTGCGTTCGGGACT-3′ for pri-miR167b, 5′-TAATACGACTCACTATAGGAACAGAAAAATCTCTCTTTCTCTTTC-3′ and 5′-GAAACAAAGTGTCTTGAAAATCAAAC-3′ for pri-miR167a, 5′-TAATACGACTCACTATAGGTTGAGTTTGAGTTTGAGTTTGAGAG-3′ and 5′-AACCTGAAGAAGATCTGGATGGAATCC-3′ for pri-miR172a, 5′-TAATACGACTCACTATAGGTCGTTTCTTGTTTTCTTTGTTTCATC-3′ and 5′-GAAAGATTGTGTAAGTGATAGAGAG-3′ for pri-miR156a, 5′-TAATACGACTCACTATAGGCTCGGACGCATATTACACATGTTC-′3 and 5′-GTCACTTAGTGGATCAAGCATGTTTTTGTGC-3′ for pri-miR319a). We resolved the products on a 5% polyacrylamide/7.5 M urea denaturing gel and excised the full-length product from the gel. RNA was phenol/chloroform extracted, ethanol precipitated, resuspended in water and stored at −80°C until use. The RNA concentration was determined based on absorbance at 260 nm using a NanoDrop 2000 Spectrophotometer (Thermo Scientific).

### *In vitro* pri-miRNA processing by DCL1

RNA was heated at 90°C for 3 min in a buffer containing 20 mM Tris–HCl (pH 7.0) and 50 mM NaCl to denature, cooled down slowly to room temperature to allow renaturation and used for subsequent experiments. Reactions were carried out at 37°C in 10 &nbsp;µl and contained 20 mM Tris–HCl (pH 7.0), 50 mM NaCl, 5 mM MgCl_2_, 1 mM ATP unless otherwise stated. Reactions were stopped by adding 10 µl of stop solution [20 mM EDTA (pH 8.0), 0.2% SDS] and ethanol-precipitated with GlycoBlue (Life Technologies). It was then dissolved in gel loading dye [95% formamide, 0.025% SDS, 18 mM EDTA (pH 8.0)] and fractionated on a 12% polyacrylamide/7.5 M urea/1× Tris/Borate/EDT (TBE) denaturing gel. The gel was stained with SYBR Gold Nucleic Acid Gel Stain (Life Technologies) and visualized using a Typhoon TRIO (GE Healthcare). The amount of 21mer and 23mer small RNA was quantified using the software Image J based on the signal intensity of a series of known amounts of the 20mer RNA oligonucleotide (5′- rArGrCrArGrUrGrGrCrUrGrGrUrUrGrArGrArUrU-3′) fractionated on the same gel (30).

### Surface plasmon resonance experiments

Surface plasmon resonance (SPR)-binding studies were carried out using a Biacore T100 (Biacore). The system was washed twice with 1× HBS-EP buffer before protein or RNA immobilization. Coupling of DCL1 (33 nM) via primary amino groups to the carboxymethyl-5 (CM5) chip was performed at a flow rate of 10 µl/min in 10 mM sodium acetate (pH 4.5) according to the manufacturer’s recommendations. The amount of coupled response unit (RU) is stated in the figure legends. For RNA coupling, a forked substrate was prepared by mixing biotinylated RNA oligo (5′-biotin-rCrArArCrCrCrArArGrArUrArGrArUrCrArUrGrCrArGrGrCrArGrCrUrUrCrArUrCrArGrArUrGrCrCrGrCrUrCrCrCrUrCrUrUrUrUrUrUrUrCrUrCrUrA-3′) with a 5-fold excess of the complementary, nonbiotinylated RNA oligo (5′-rGrCrUrCrCrCrUrCrUrUrUrUrUrUrUrCrUrCrUrArGrGrCrArUrCrUrGrArUrGrArArGrCrUrGrCrCrUrGrCrArUrGrArUrCrUrArUrCrUrUrGrGrGrUrUrG-3′) in a buffer containing 20 mM Tris–HCl (pH 7.0) and 50 mM NaCl and annealed by heating at 90°C for 3 min and slowly cooling down to room temperature. An S-series streptavidin (SA) sensor chip was activated by three regeneration injections of (50 mM NaOH and 1 M NaCl) followed by immobilization of the biotinylated RNA at a flow rate of 10 µl/min; the coupled RU are given in the figure legends. A control flow cell was activated and blocked in the absence of protein or RNA was used to subtract the RU resulting from the non-specific interaction with the sensor chip and the buffer’s bulk refractive index. Before conducting the binding study, the system was primed twice with the corresponding binding buffer. To detect the interaction of DCL1 and SE with RNA at 50 mM and 150 mM NaCl concentrations, each protein at a concentration of 43 nM was injected at a flow rate of 20 µl/min for 70 s in a binding buffer containing 20 mM Tris–HCl (pH 7.0), 50 mM or 150 mM NaCl and 2 mM &nbsp;β-ME. To detect the interaction of SE and its mutant variants, each protein at a concentration of 20 nM was injected at a flow rate of 20 µl/min for 70 s in a binding buffer containing 20 mM Tris–HCl (pH 7.0), 50 mM NaCl, 0.005% surfactant P20 and 2 mM β-ME. The chip surface was regenerated by three 100 µl of injections of buffer containing 1.5 M NaCl at a flow rate of 100 µl/min. To detect the interaction of SE with the DCL1/RNA complex, DCL1 at a concentration of 16 nM was first injected until ∼1200 RU of DCL1 bound to the RNA, and then wild-type SE protein was injected as described earlier in the text. After the chip surface regeneration, the aforementioned steps were repeated for the mutant SE proteins. Because the RU of DCL1 bound to RNA was slightly different for each SE protein injection, the RU obtained by mutant SE protein injection was normalized based on the ratio of the RU of DCL1 bound to RNA for wild-type SE protein to that for the corresponding mutant SE protein. The relationship between the amount of coupled DCL1 or RNA (RU*_ligand_*) and the bound protein (RU*_analyte_*) and their stoichiometry were determined based on the following equation:
(1)
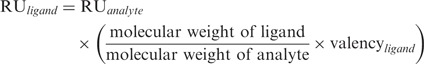



## RESULTS

### Optimization of pri-miRNA processing by DCL1

**Figure 1.**
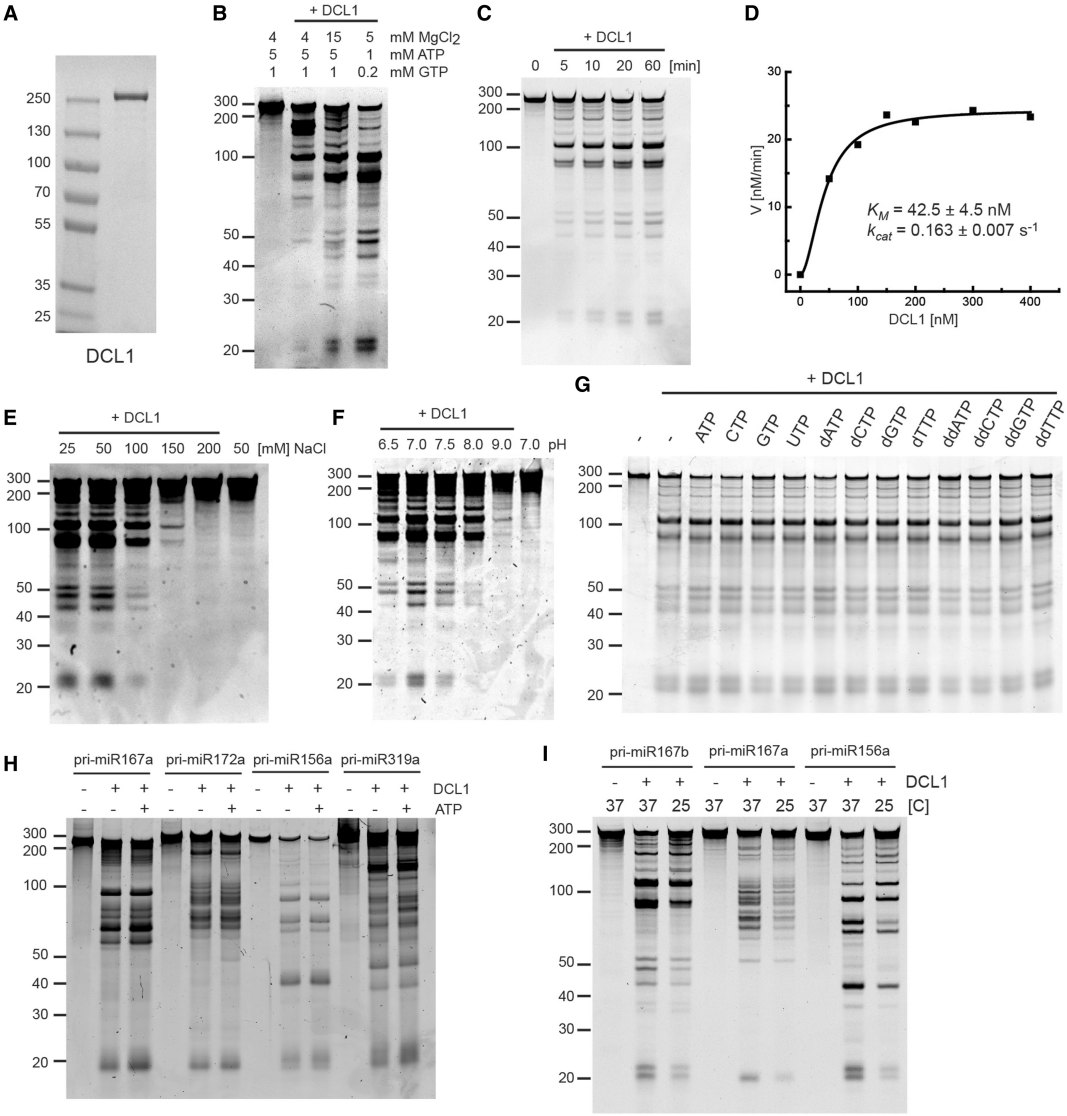
Characterization of pri-miRNA processing by DCL1. (**A**) Purified recombinant DCL1 protein. Coomassie blue-stained SDS–PAGE of purified DCL1 expressed in a baculovirus/insect cell expression system. (**B**) pri-miR167b processing by DCL1. The reaction was carried out under the same conditions as described previously (29) and at different concentrations of MgCl_2_, ATP and GTP. Pri-miR167b (150 nM) and DCL1 (50 nM) were incubated in a buffer [20 mM Tris–HCl (pH 7.0), 50 mM NaCl and indicated concentrations of MgCl_2_, ATP and GTP] at 37°C for 60 min. RNA was fractionated on 12% polyacrylamide/7.5 M urea denaturing gel and visualized by SYBR Gold staining. (**C**) Time course analysis of pri-miR167b processing. Pri-miR167b (150 nM) and DCL1 (50 nM) were incubated in a buffer [20 mM Tris–HCl (pH 7.0), 50 mM NaCl, 5 mM MgCl_2_ and 1 mM ATP] at 37°C for the indicated times. RNA was fractionated and detected as in (B). (**D**) pri-miR167b processing with a varying concentration of DCL1. The reaction was done at 37°C for 7 min with the same buffer components as in (C). The RNA was fractionated and detected as in (B). The amount of small RNA with 21mer and 23mer lengths was determined based on a series of known amounts of 20mer RNA fractionated alongside, and the velocity was plotted as a function of DCL1 concentration. (**E**) Effect of NaCl concentration. Pri-miR167b (150 nM) and DCL1 (50 nM) were incubated in a reaction buffer [20 mM Tris–HCl (pH 7.0), 5 mM MgCl_2_, 1 mM ATP and the indicated concentrations of NaCl] at 37°C for 20 min. The RNA was fractionated and detected as in (B). (**F**) Effect of pH. Pri-miR167b (150 nM) and DCL1 (50 nM) were incubated in a reaction buffer (50 mM NaCl, 5 mM MgCl_2_, 1 mM ATP and 20 mM Tris–HCl at the indicated pH) at 37°C for 20 min. The RNA was fractionated and detected as in (B). (**G**) Effect of nucleoside triphosphate. Pri-miR167b (150 nM) and DCL1 (50 nM) were incubated in a reaction buffer [20 mM Tris–HCl (pH 7.0), 50 mM NaCl, 5 mM MgCl_2_, and the indicated nucleoside triphosphates at 1 mM] at 37°C for 60 min. The RNA was fractionated and detected as in (B). (**H**) Effect of ATP on four pri-miRNA substrates. The reaction was done with the indicated pri-miRNA (150 nM) and DCL1 (50 nM) with or without 1 mM ATP as in (G). The RNA was fractionated and detected as in (B). (**I**) Effect of temperature. The reaction was done with the indicated pri-miRNA (150 nM) and DCL1 (50 nM) in a reaction buffer [20 mM Tris–HCl (pH 7.0), 5 mM MgCl_2_, 1 mM ATP and 50 mM NaCl] at 37°C or 25°C for 20 min. The RNA was fractionated and detected as in (B).

DCL1 was N-terminally epitope-tagged with SUMOstar tag, a hexahistidine tag and Strep-tag II and expressed in a baculovirus insect cell expression system. After a two-step affinity purification using Ni-NTA and StrepTactin columns, the SUMOstar tag was removed by incubating with SUMOstar protease. The resulting DCL1 protein does not bear any extra amino-acid residues at its N-terminal end, which eliminates the possibility that the epitope tag interferes with its function. The untagged DCL1 was further purified by gel filtration chromatography (Figure 1A).

It was previously shown that DCL1 alone is able to cleave transcribed pri-miR167b and excise miRNA/miRNA* duplex *in vitro* (29). However, the reaction used had an excess of ATP/GTP over magnesium ions, leaving little free magnesium for the RNaseIII activity of DCL1 (29). We therefore carried out the reaction with different ratios of MgCl_2_ and ATP/GTP at different concentrations and measured DCL1 activity by the release of miRNA/miRNA*. Although some release of small RNA of the expected sizes for miRNA and miRNA* (21 and 23, respectively) from the pri-miRNA was observed under the previously described conditions (4 mM MgCl_2_, 5 mM ATP and 1 mM GTP), significant enhancement in the processing reaction was observed when the MgCl_2_ concentration was increased to 15 mM, and a further enhancement was observed by decreasing both the MgCl_2_ and ATP/GTP concentrations while maintaining an excess of Mg^2+^ over the nucleotides (Figure 1B).

We next carried out a basic enzymatic characterization of DCL1 with pri-miR167b under the conditions of excess Mg^2+^. A time course study under excess-substrate conditions showed that small RNA accumulates linearly in the first 10 min and reaches a plateau during the 60 min reaction (Figure 1C). The apparent *K*_M_ and *k*_cat_ determined by quantifying the small RNA accumulation as a function of increasing DCL1 concentration in the linear time range were 42.5 ± 4.5 nM and 0.163 ± 0.007 s^−^^1^, respectively (Figure 1D). As shown in the previous report using dsRNA substrate (29), DCL1 activity on pri-miR167b also decreased on increasing NaCl concentration (Figure 1E). We further found that DCL1 activity is sensitive to pH, with maximal activity at pH 7.0 (Figure 1F). The pattern of the processed fragments was not affected by varying either the NaCl concentration or the pH (Figure 1E and F).

We next investigated the effect of nucleoside triphosphates on DCL1 activity. We observed that DCL1 activity on pri-miR167b was only slightly stimulated by nucleoside triphosphates, including ATP and GTP, as well as by deoxynucleoside- or dideoxynucleoside-triphosphates (Figure 1G). Nucleoside triphosphate (NTP)-independent activity was also observed with four other pri-miRNA substrates, although a modest stimulation was observed with some substrates (Figure 1H).

Finally, we tested the effect of temperature on DCL1 activity, as the optimal temperature for Arabidopsis growth is 23–25°C. More importantly, temperature may have a direct effect on RNA secondary structure and therefore on the specificity of DCL1 cleavage reaction. Decreasing the reaction temperature from the standard 37–25°C reduced the efficiency of the processing reaction and subsequent small RNA accumulation, but it did not affect the pattern of the fragments produced by processing of three different pri-miRNA substrates (Figure 1I).

### The DCL1 processing reaction is stimulated by SE in an ionic strength-dependent manner

**Figure 2.**
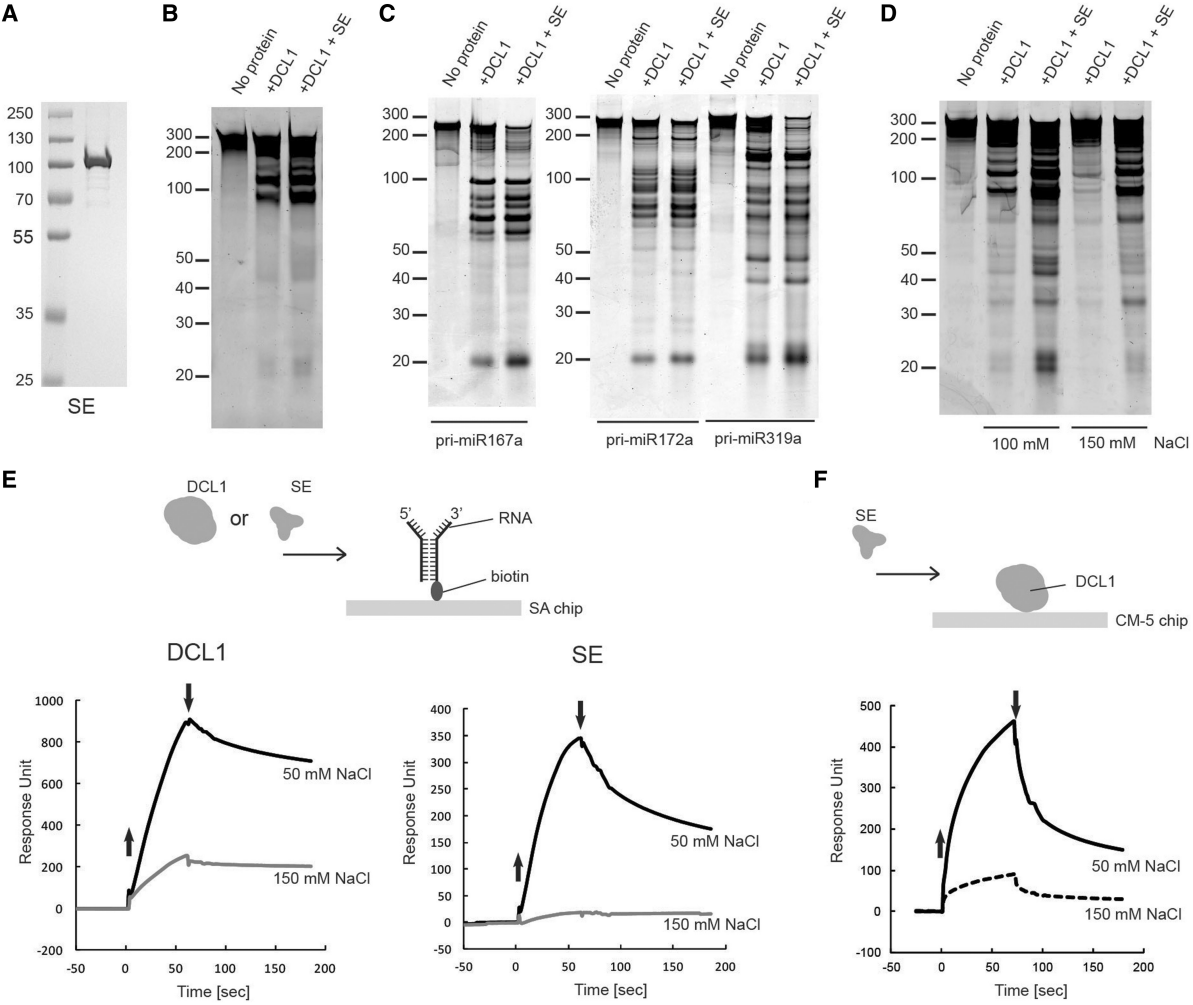
Effect of SE on pri-miRNA processing. (**A**) Purified recombinant SE protein. Coomassie blue stained SDS–PAGE of purified SE expressed in a baculovirus/insect cell expression system. (**B**) Effect of SE on pri-miR167b processing by DCL1. Pri-miR167b (150 nM) was incubated with DCL1 (50 nM) and SE (240 nM) in a reaction buffer [20 mM Tris–HCl (pH 7.0), 50 mM NaCl, 5 mM MgCl_2_ and 1 mM ATP] at 37°C for 20 min. The RNA was fractionated on 12% polyacrylamide/7.5 M urea denaturing gel and visualized by SYBR Gold staining. (**C**) Effect of SE using three different pri-miRNA substrates. The reaction was done as in (B) with the indicated pri-miRNA substrates (150 nM). The RNA was fractionated and visualized as in (B). (**D**) Effect of SE on DCL1 at different NaCl concentrations. The reaction was done as in (B) with pri-miR167b, and the RNA was fractionated and visualized as in (B). (**E**) SPR analysis of interaction of DCL1 and SE with RNA. In all, 240 RU of biotin-labeled forked-RNA consisting of a 40-base pairs dsRNA region with a forked structure on one end that consist of a 20-base ssRNA on both strands was immobilized on the SA-coated sensor chip as shown in the schematic diagram, and DCL1 or SE was introduced for 70 s at a concentrations of 42 nM, in a buffer containing 20 mM Tris–HCl (pH 7.0), 2 mM β-ME, and either 50 mM or 150 mM NaCl. The up and down arrows indicate the start and end of protein injections, respectively. (**F**) SPR analysis of interaction of SE with DCL1. 1750 RU of DCL1 was immobilized via its amine group on the CM-5 sensor chip, and SE was injected for 70 s at a concentration of 20 nM in a buffer containing 20 mM Tris–HCl (pH 7.0), 2 mM β-ME, 0.005% surfactant P20 and 50 mM or 150 mM NaCl. The up and down arrows indicate the start and end of protein injections, respectively.

It was previously reported that both the efficiency and accuracy of *in vitro* pri-miRNA processing by DCL1 are enhanced by recombinant hexahistidine-tagged HYL1 and SE proteins that are expressed in insect cells and purified by using metal affinity chromatography (29). As the effect of SE on the processing reaction *in vitro* is much more pronounced than that of HYL1 (29), we focus our efforts on elucidating the function of SE on pri-miRNA processing reaction. To simplify the purification scheme and eliminate the possibility of functional interference by the extra amino acid sequence at the ends of SE, we expressed recombinant SE as the N-terminally SUMOstar-tagged forms and purified its untagged form as described earlier in the text for DCL1 (Figure 2A). As shown in Figure 2B, SE stimulated pri-miR167b processing by 1.5- ± 0.3-fold. Stimulation of DCL1 activity by SE was also observed with three additional but different pri-miRNA substrates (Figure 2C).

**Figure 3.**
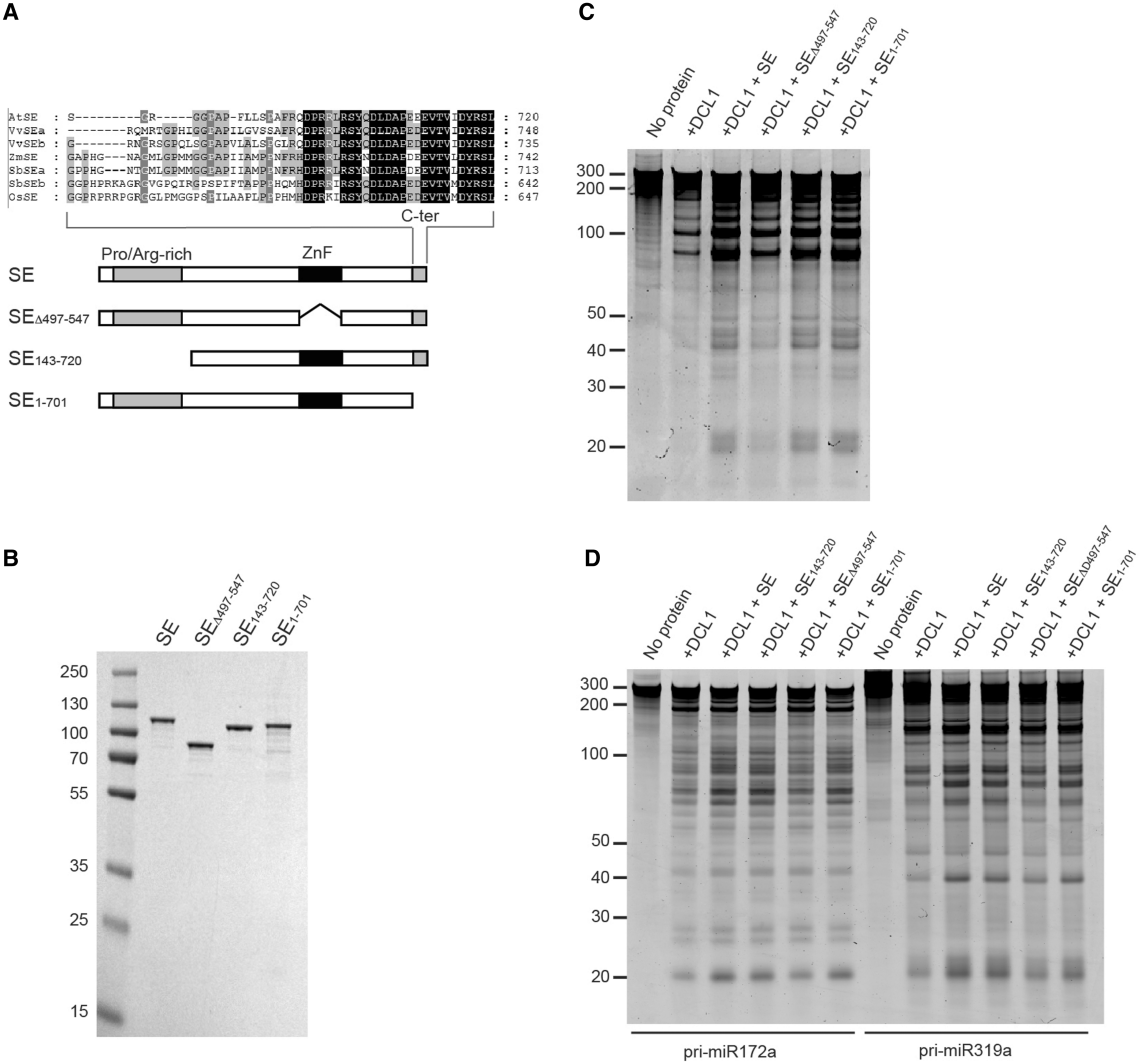
Effect of SE and its mutant variants on pri-miRNA processing by DCL1. (**A**) Schematic representation of the SE domain structure and SE mutant proteins used in the following experiments. The N-terminal, proline/arginine-rich region (Pro/Arg-rich), ZnF domain and the C-terminal region (C-ter) are indicated. The inset shows the alignment of the C-ter end from plant SE homologs (amino acid residues conserved in four or more SE species are shaded in gray, and those conserved in all SE species are shaded in black. At; Arabidopsis thaliana, Vv; Vitis vinifera, Zm; Zea mays, Sb; Sorghum bicolor, Os; Oryza sativa). The mutant SE proteins, in which ZnF, N-terminal, and C-terminal domains are deleted, are designated as SE_Δ497–547_, SE_143–720_ and SE_1–701_, respectively. (**B**) Purified recombinant SE and its mutant proteins. Coomassie blue-stained SDS–PAGE of purified SE proteins expressed in a baculovirus/insect cell expression system. (**C**) Effect of SE and its mutant variants on pri-miR167b processing. Pri-miR167b (150 nM) was incubated with DCL1 (50 nM) and wild-type SE and its deletion mutants (240 nM) in a reaction buffer [20 mM Tris–HCl (pH 7.0), 100 mM NaCl, 5 mM MgCl_2_ and 1 mM ATP] at 37°C for 20 min. The RNA was fractionated on a 12% polyacrylamide/7.5 M Urea denaturing gel and visualized by SYBR Gold staining. (**D**) Effect of SE and its mutant variants on DCL1’s processing reaction on two more pri-miRNA substrates. The reaction and detection were done as in (C) with the indicated pri-miRNA substrates (150 nM). The RNA was fractionated and visualized as in (C).

To explore why the effect of SE on the processing reaction was moderate (Figure 2B and C), we ran the reaction under more physiologically relevant salt concentrations. As shown in Figure 2D, stimulation of small RNA accumulation by SE was greater at the higher salt concentration. We observed a 4.0-fold stimulation of small RNA accumulation by SE at 100 mM NaCl versus 2.0-fold stimulation at 50 mM NaCl, whereas limited amount of small RNA accumulation was observed in the absence of SE at 150 mM NaCl. However, the effect of SE on DCL1 activity displayed some substrate specificity, as we observed with pri-miR172a and pri-miR319a a 2- to 3-fold stimulation at 100 mM NaCl (shown below in Figure 3D).

To understand the basis for the salt-dependent SE stimulation of DCL1 activity, we first examined the ability of DCL1 and SE to directly interact with RNA at different salt concentrations using SPR. We immobilized a biotin-labeled RNA that consists of a 40 bp dsRNA region with a forked structure on one end that consist of a 20-base ssRNA on both strands (termed forked-RNA) on a SA-coated sensor chip and then injected DCL1 or SE proteins. The forked-RNA was designed after pri-miR167a, whose processing was stimulated by SE *in vitro* (Figure 2C) and *in vivo* (5). Furthermore, DCL1 processes pri-miRNA mainly from a forked-RNA structure with few exceptions from a loop structure. At 50 mM NaCl concentration, both proteins were able to bind to RNA as evidenced by the increase in RUs at the start of protein injection (Figure 2E). SE showed weaker RNA binding, as judged by its rapid dissociation at the end of protein injection and during the wash with buffer (Figure 2E). The interaction of DCL1 and SE with RNA is electrostatic in nature as shown by its sensitivity to salt concentration. At 150 mM NaCl, the maximum RU (RU_max_) of bound DCL1 or SE that was reached before the start of the wash with buffer decreased by 4- and 18-fold, respectively, with nearly negligible binding of SE to RNA (Figure 2E). We next asked whether the purified SE binds directly to DCL1 *in vitro*. We first immobilized DCL1 via its amine groups on a CM5 sensor chip and then injected SE (Figure 2F). SE bound to DCL1 in an ionic strength-dependent manner with a 5-fold reduction in RU_max_ on increasing the NaCl concentration from 50 to 150 mM (Figure 2F). The ability of SE to interact with DCL1 but not with RNA at higher salt concentration suggests that SE binding to RNA might not be essential for stimulating DCL1 activity.

### The ZnF domain of SE is critical for SE stimulation of pri-miRNA processing by DCL1

Our observation that SE stimulates DCL1 activity prompted us to ask what part or parts of the SE are required for its stimulatory effect. The only SE domain readily identifiable by a protein homology search was its ZnF domain. We therefore did a sequence alignment analysis to detect homologous amino acid sequences among SE proteins from several plant species (Figure 3A). We detected a conserved low complexity region at the N-terminus (amino acids 7–139) containing 30 proline and 30 arginine residues. We also found a conserved amino acid region at the extreme C-terminus (amino acids 695–720). The conservation among these regions of plant SE proteins suggests functional significance. The importance of the C-terminal domain is also suggested by the observation that miRNA accumulation is compromised in the *se-1* mutant in which the C-terminal domain is disrupted by a 7 bp deletion located immediately before the stop codon of the coding sequence (31). To ask whether the ZnF domain and the conserved regions at both ends are required for SE’s stimulatory effect on pri-miRNA processing reaction, we made N-terminally hexahistidine-tagged expression constructs with N-terminal, C-terminal or the ZnF domain deletions, as well as of the wild-type SE protein. We designated them SE_143__–720_, SE_1__–701_, and SE_Δ497__–547_, respectively (Figure 3A and B).

We assessed the ability of these SE mutant variants to stimulate DCL1’s processing activity. His-tagged SE stimulated DCL1’s activity at a similar level as the untagged SE (Figure 3C cf. Figure 2D). SE_Δ497__–547_ did not stimulate small RNA accumulation, whereas SE_1__–701_ and SE_142__–720_ were still able to do so (Figure 3C), indicating that the ZnF domain is important for SE’s ability to enhance DCL1’s cleavage reaction while that of the N-terminal or C-terminal domain is dispensable. The same trend was also observed with two other pri-miRNA substrates (Figure 3D).

### Direct binding of SE to DCL1 is compromised by a deletion in either the N-terminal or the ZnF domain

**Figure 4.**
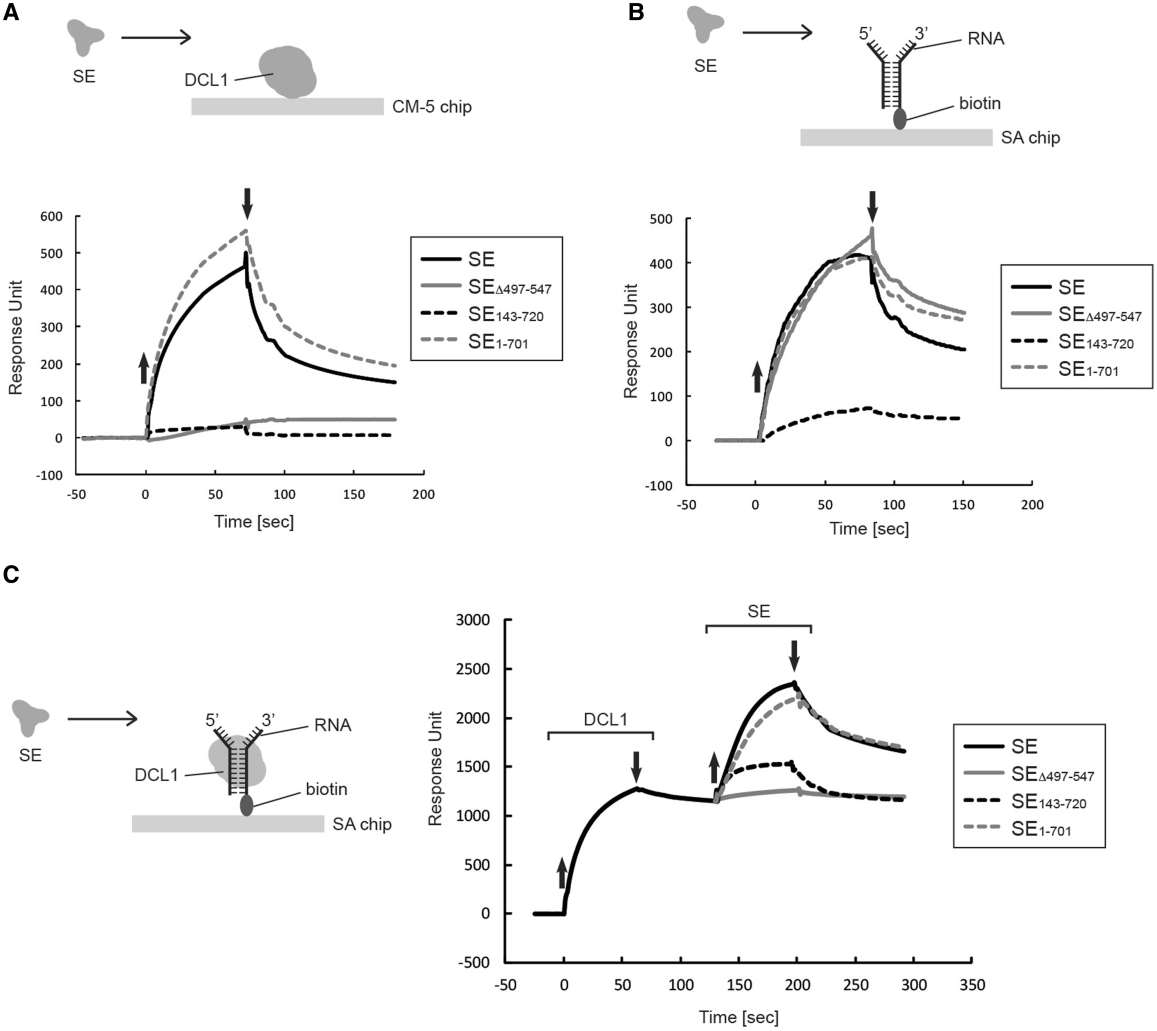
Interaction of SE and its mutant variants with DCL1 and/or RNA. (**A**) Interaction of SE and its mutant variants with DCL1. Approximately 1750 RU of DCL1 was immobilized via its amine group on the CM-5 sensor chip and SE protein was injected for 70 s in a buffer containing 20 mM Tris–HCl (pH 7.0), 50 mM NaCl, 2 mM β-ME and 0.005% surfactant P20. The concentration of SE and its mutants was 20 nM. The up and down arrows indicate the start and end of protein injection, respectively. (**B**) Interaction of SE and its mutant variants with RNA. Approximately 210 RU of RNA was immobilized as in Figure 2E, and SE and its mutant variants were injected in a buffer containing 20 mM Tris–HCl (pH 7.0), 50 mM NaCl, 2 mM β-ME and 0.005% surfactant P20 at concentration of 20 nM. The up and down arrows indicate the start and end of protein injections, respectively. (**C**) Interaction of SE and its mutant variants with DCL1 in the presence of RNA. Approximately 1200 RU of DCL1 was bound to 60 RU of biotin-labeled RNA immobilized on the SA-coated sensor chip as in Figure 2E, and then SE and its mutant variants were injected for 70 s as shown in the diagram in a buffer containing 20 mM Tris–HCl (pH 7.0), 50 mM NaCl, 2 mM β-ME and 0.005% surfactant P20 at a concentration of 20 nM. The up and down arrows indicate the start and end, respectively, of injections of either DCL1 or SE.

To understand the mechanism by which SE stimulates DCL1’s processing reaction through its ZnF domain, we carried out protein–protein and protein–RNA-binding assays using SPR. We first assessed the direct interaction of SE and its mutant variants with DCL1 immobilized on a CM5 chip. The C-terminally truncated SE_1__–701_ bound to DCL1 as well as did SE in low salt (50 mM NaCl) (Figure 4A). By contrast, neither the N-terminally truncated SE_143__–720_ nor SE_Δ497__–547_ lacking the ZnF domain bound to DCL1 under these salt conditions (Figure 4A). These results are surprising, as SE_143__–720_ stimulated DCL1 activity, despite its inability to interact directly with DCL1.

### Direct binding of SE to RNA requires the N-terminal domain, but not the ZnF domain

We next assessed the direct interaction of SE and its mutant variants with forked-RNA immobilized on a SA-coated chip as described earlier in the text. At 50 mM NaCl concentration, SE_1__–701_ and SE_Δ497__–547_ interacted with RNA as did SE, whereas SE_143__–720_ showed a 6-fold reduction in its interaction with RNA (Figure 4B), revealing that SE primarily binds to RNA through its N-terminal sequence with minimal contribution from other regions. These results are unexpected and raise an interesting paradox as to how SE_143__–720_ stimulates DCL1 activity without directly interacting with DCL1 or with RNA.

### RNA promotes interactions between DCL1 and SE and stimulates DCL1’s activity

We next asked whether SE and its variants could interact with DCL1 bound to RNA in an attempt to understand why SE_143__–720_ and not SE_Δ497__–547_ stimulated DCL1 activity, despite their inability to interact directly with DCL1. We first immobilized forked-RNA on a SA-chip and injected DCL1 to form a DCL1/RNA complex. This binding study was also performed at 50 mM NaCl using a saturating DCL1 concentration to ensure saturation of the entire surface-tethered RNA and preassembly of a stable DCL1/RNA complex.

Roughly 1200 RU of DCL1 (214 kDa) remained tightly bound to the 60 RU immobilized RNA (39 kDa) before the injection of SE; we estimated based on Equation (1) in ‘Materials and Methods’ section that DCL1 bound RNA with a nearly 2.8:1 molar ratio under these conditions (Figure 4C). When SE (hexahistidine-tagged SE, 86 kDa) was added to the DCL1/RNA complex (638.2 kDa), a further 1150 RU of DCL1 was accumulated (Figure 4C); the stoichiometry of SE to DCL1 was estimated to be 2.4:1. SE interacted with DCL1 alone or with the DCL1/RNA complex with similar affinity as evidenced by the similar dissociation kinetics of the bound SE during the wash with buffer. However, the stoichiometry of the binding was strikingly different. Under the same experimental conditions, SE bound DCL1 with a 0.7:1 molar ratio (Figure 4A). This suggests that binding of DCL1 to RNA triggers a conformational change within DCL1 to expose more binding sites to interact with SE. SE_1__–701_ interacted with the DCL1/RNA complex as well as did the intact SE protein (Figure 4C). Surprisingly, SE_143__–720_ (69 kDa), which did not interact with DCL1 or RNA alone, was able to bind to the DCL1/RNA complex (Figure 4C). However, this binding was weaker than with SE, as 2-fold less RU_max_ accumulated during protein injection and the bound SE_1__–701_ dissociated at a faster rate during the wash with buffer. This weaker binding is nonetheless sufficient to stimulate DCL1’s processing activity (Figure 3C and D). Lastly, SE_Δ497__–547_ did not bind to DCL1 even in the presence of RNA (Figure 4C). The fact that SE_Δ497__–547_ is able to bind to RNA directly (Figure 4B) provides an additional control that under our experimental condition all of the surface-immobilized RNA molecules are pre-bound to DCL1 prior to the injection of SE proteins.

## DISCUSSION

In the present study using highly purified proteins, we find that SE substantially enhances processing of the pri-miRNA under optimized reaction conditions. We showed that SE interacts directly with DCL1 and with RNA. We further showed that SE interacts with RNA through its conserved N-terminal proline/arginine-rich domain with minimal contribution from other regions and that both the N-terminal region and the conserved ZnF domain of SE are required for its interaction with DCL1 protein alone. However, SE lacking the N-terminal sequence can bind to RNA-bound DCL1 and stimulate processing, but SE lacking its ZnF domain cannot. Thus, although the N-terminal domain of SE appears to be required for RNA binding, the ability of the N-terminally truncated SE protein to stimulate DCL1 activity suggests that it does so directly and that RNA binding is not required for this stimulation.

We first carried out enzymatic characterization of DCL1 activity alone on several pri-miRNA substrates and identified its optimal reaction conditions. Interestingly, despite the presence of a helicase domain in DCL1, nucleotide binding and hydrolysis is generally not required for DCL1 activity, and when stimulation is observed, it is modest (Figure 1G and H). The helicase domain in DCL1 and other Dicers from plants and animals belongs to the RIG-I family of RNA helicases that exhibit robust ATP-dependent translocation activity on dsRNA (32). However, different Dicers display either ATP-dependent or ATP-independent processing activity. *Drosophila* Dicer-2 (33) and *Caenorhabditis elegans* DCR-1 exhibit ATP-dependent cleavage of dsRNA (34), whereas human Dicer and *Drosophila* Dicer-1 cleave substrate RNA without a requirement for ATP (35–37). Plant DCL1 is a unique Dicer, as it performs the first cleavage of pri-miRNA internally, whereas most other Dicer enzymes cleave dsRNA and pre-miRNA from their open helical ends (38). Considering the role of some Dicer helicase domains in recognizing the structural feature embedded in RNA substrates (34,39), it is conceivable that the helicase domain of DCL1 is required for recognizing certain structural features in pri-miRNA. Recent work shows that the ATP- and helicase dependencies of DCL1 vary among different pri-miRNA substrates (40). We also observed some degree of ATP dependence with some pri-miRNA substrates, but not with others. We therefore predict variation in the extent to which DCL1 requires the helicase domain to recognize different RNA substrates, but that generally DCL1 processes pri-miRNA in an ATP-independent manner.

The DCL1 processing reaction is highly salt concentration dependent, and at 150 mM NaCl, a physiologically relevant salt concentration, we observed little pri-miRNA cleavage and release of small RNA (Figures 1E and 2D). This indicates that despite the intrinsic ability of DCL1 to recognize structural features embedded in pri-miRNA, other factors are required to cleave pri-miRNA and to release the miRNA/miRNA* duplex. We find that DCL1 becomes more dependent on SE at a high ionic strength (Figure 2D), although this dependence varies among pri-miRNA substrates (Figure 3D). These results are consistent with genetic evidence that *se* mutants display less miRNA and more pri-miRNA accumulation than do wild-type plants. We therefore conclude that SE is integral to DCL1-dependent pri-miRNA processing and suggest that this interaction may regulate the miRNA accumulation in the cell. The substrate-dependent stimulation of DCL1 activity by SE might also provide a mechanism for the selective accumulation of specific miRNAs.

We used deletion mutants of SE to study the mechanism by which SE interacts with RNA and stimulates the activity of DCL1. SE primarily uses its N-terminal region to interact with RNA, as evidenced by the marked reduction in RNA binding by SE_143__–720_ (Figure 4B). Indeed, the N-terminal domain (amino acids 1–142) is highly basic, containing 31 arginine residues, although there may be other part(s) of SE that contributes to SE’s ability to bind to RNA. The importance of the N-terminal domain in RNA binding can be inferred from the previous observation that SE core + C, which corresponds to amino acids 194–720 and resembles SE_143__–720_, exhibits only weak affinity for RNA and that it requires a concentration of ∼83 µM to reach 1:1 binding (28). Both the N-terminal region and the ZnF domain are required for the interaction of SE with DCL1 when DCL1 is not bound to RNA (Figure 4A). It is not clear how both sites are critical at once, but a plausible mechanism would be that binding to one site triggers a conformational change in DCL1 leading to binding of the other domain. Interestingly, SE_143__–720_ was able to recover a significant portion of its interaction with DCL1 in the presence of RNA (Figure 4C), demonstrating that the ZnF domain binds to DCL1 and that the N-terminal region contributes to SE binding to the DCL1/pri-miRNA complex. Nonetheless, the interaction with the ZnF domain is sufficient to stimulate full DCL1 activity, as evidenced by the equal stimulation observed with SE_143__–720_ and with the wild-type SE (Figure 3C and D). It is also not clear whether DCL1 uses the same binding sites to interact with SE in the presence or absence of RNA. We anticipate that when DCL1 binds to RNA, it exposes more binding sites for SE, as indicated by the increase in its binding stoichiometry to DCL1 when DCL1 is bound to RNA (Figure 4A and C). This raises the possibility that the N-terminal and the ZnF regions bind to different sites on DCL1 when DCL1 is bound to RNA and that binding via the ZnF domain is catalytically critical. The ability of SE_143__–720_, but not SE_Δ497__–547_, to stimulate DCL1 processing activity demonstrates that binding of SE to RNA is not required to stimulate DCL1 activity. This is also supported by the observation that SE stimulates DCL1 activity at 150 mM NaCl concentration, where SE does not bind to RNA. Taken together, these observations suggest a model in which DCL1 binding to RNA initiates conformational changes in DCL1 that, in turn, initiate a catalytically critical interaction with the ZnF domain of SE. SE might be oriented such that the ZnF domain and perhaps a portion of the N-terminal region are bound to DCL1, whereas the N-terminal region is bound to RNA. We do not know the identity of the sequence within the DCL1 protein that interacts with the ZnF domain of SE, nor how this interaction stimulates DCL1 activity. DCL1 can bind well to RNA at a high salt concentration (Figure 2E); yet, salt inhibits its cleavage activity significantly (Figures 1E and 2D). This suggests that although DCL1 is bound to RNA, the RNaseIII domains at the active site are not properly positioned to mediate efficient cleavage. We propose that the ZnF domain binds near or at the active site of DCL1 and enhances the assembly of the two active-site RNaseIII domains with RNA. Although we demonstrated that the ZnF domain is dispensable for the interaction of SE with RNA, it remains possible that the binding of DCL1 to RNA could also promote a conformational change in the RNA that is specifically tailored to interact with the ZnF domain. It is not clear whether SE stimulates the first, the second or both cleavages of pri-miRNA by DCL1, particularly because SE stimulates DCL1 activity in substrate specific manner (Figure 3D).

There are a number of additional candidate genes that are likely to function during miRNA biogenesis (10–17). The most promising candidate would be CBC, which binds to the 5′ cap structure of pri-miRNAs and protein-coding mRNAs and has been shown to be involved in miRNA biogenesis. We predict that the highly conserved C-terminal domain of SE (Figure 3A), which is not involved in binding or stimulating DCL1 activity, is required for its interaction with CBC. Phenotypes of *se-1* mutant plants, in which the C-terminal domain is disrupted by a 7 bp deletion immediately before the stop codon of the *SE* gene (31), are reminiscent of mutants that are defective in CBC (10). Indeed, the recent report demonstrated that a component of CBC interacts with SE (41). The functional connection between SE and CBC can also be inferred from animal studies, which showed that Ars2, an animal homolog of SE, co-immunoprecipitates with CBC, and its knockdown results in reduced miRNA accumulation in flies and mice (42,43). Inclusion of one or more of the candidate proteins that *in vivo* studies suggest to play a role in pri-miRNA biogenesis may be critical for the reconstitution of the full miRNA biogenesis reaction *in vitro* and a further understanding of its recognition and cleavage mechanisms.

## FUNDING

King Abdullah University Startup Allowance. Funding for open access charge: Laboratory Base Line Funds.

*Conflict of interest statement*. None declared.
